# Cardiovascular Disease After COVID-19: A Call to Action∗

**DOI:** 10.1016/j.jacadv.2023.100448

**Published:** 2023-08-03

**Authors:** Leah B. Kosyakovsky, Federico Angriman

**Affiliations:** a Division of Cardiology, Beth Israel Deaconess Medical Center, Harvard Medical School, Boston, Massachusetts, USA; b Department of Critical Care Medicine, Sunnybrook Health Sciences Centre, Toronto, Ontario, Canada; c Interdepartmental Division of Critical Care Medicine, University of Toronto, Toronto, Ontario, Canada

**Keywords:** COVID-19, CVD, MI

**Figure:**
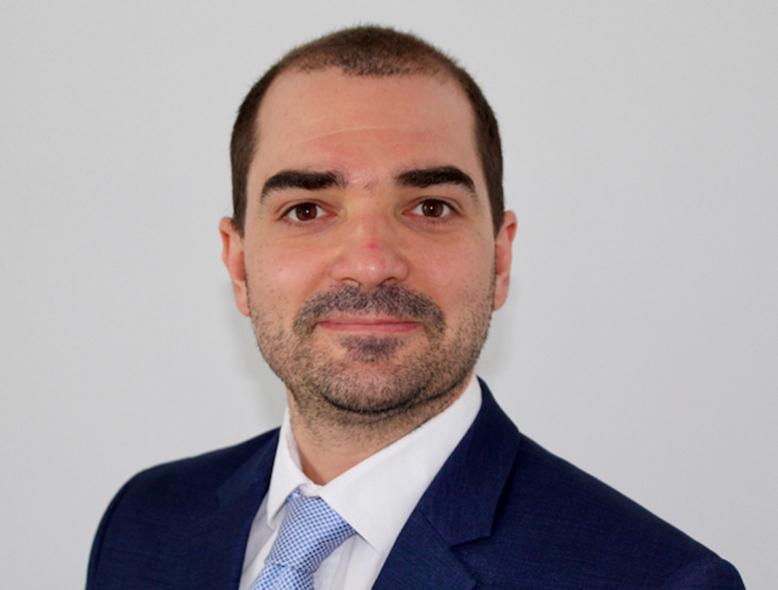

The impact of viral and bacterial infections on subsequent cardiovascular disease (CVD) risk has been established, although the exact mechanisms and underpinnings of this increased risk remain elusive.^1^, ^2^, ^3^, ^4^ The COVID-19 pandemic showcased the relevance of long-term infectious sequelae, with particular emphasis on cardiovascular complications.^5^, ^6^, ^7^, ^8^, ^9^, ^10^ The pandemic represents a uniquely apt scenario to better understand the relationship between infection and subsequent CVD, through the lens of a single, (now) well-known pathogen with meticulously studied host-pathogen interactions.^6^ Given the overwhelming number of patients with past COVID-19 infection, even small changes in long-term CVD may be very relevant from a population perspective and may also signify an unmet need in mitigation.

In this issue of *JACC: Advances*, Zahid et al^11^ present the results of a large nationwide study evaluating the incidence of myocardial infarction (MI) at 30 days after an index hospitalization for COVID-19. The authors used data from the Nationwide Readmission Database during 2020; their primary outcome was the incidence of 30-day readmission due to a MI. They included 521,351 patients with a COVID-related hospitalization, reporting a 0.6% risk of MI at 30 days. MI represented approximately 5% of all hospital readmissions, and type 2 events were more frequent than type 1 events. Several characteristics were associated with a higher odds of rehospitalization for MI, including male sex, increasing age, and increasing burden of comorbidity. Notably, higher income was associated with a lower odds of MI readmission. Additionally, a concomitant admission diagnosis of COVID-19 among patients readmitted with MI within 30 days was associated with increased readmission mortality.

The study by Zahid et al^11^ has several strengths. First, the authors should be congratulated for completing a large, relevant study during an extremely challenging time. The use of a large, nationwide cohort increases the generalizability of its findings. Furthermore, the study provides precise estimates of readmission risk and incidence of MI after COVID-19, which are comparable (or lower) to previous estimates after infection, sepsis, or COVID-19 (Table 1).^2^^,^^5^^,^^12^^,^^13^ These estimates can be utilized to better inform care after a COVID-19 hospitalization, including the development of strategies to mitigate the risk of post-COVID CVD. This study also provides important information regarding risk factors for CVD after COVID-19, thus informing potential future studies around CVD prevention. These data also highlight the importance of prior comorbid trajectory and social determinants of health in the long-term outcome pathway after a hospitalization for infection.^14^ Prior studies have shown similar relationships between comorbid characteristics and cardiovascular events in a general population of patients with sepsis of varying etiology.^15^, ^16^, ^17^

**Table 1:** Summary of Selected Studies Evaluating Cardiovascular Outcomes After COVID-19

First Author (Year)	Population	Main Outcomes	Summary of Findings
Xie et al^5^ (2022)	U.S. veterans who survived the first 30 d of COVID-19; compared to nonhospitalized and hospitalized non-COVID-19 cohorts	Cerebrovascular disorders, dysrhythmia, inflammatory heart disease, ischemic heart disease, thrombotic disorders	Increased risk of cardiovascular disease (up to 1 y)
Raisi-Estabragh et al^8^ (2023)	COVID-19 cases; propensity score-matched to 2 uninfected controls	Myocardial infarction, stroke, heart failure, atrial fibrillation, venous thromboembolism, pericarditis, all-cause death, cardiovascular death	Increased risk of cardiovascular outcomes and all-cause mortality (average follow-up 141 d)
Wang et al^9^ (2022)	Unvaccinated survivors of COVID-19; propensity score-matched to survivors without COVID-19 (not necessarily hospitalized)	Stroke, arrhythmia, inflammatory heart disease, ischemic heart disease, thrombotic disorders	Increased risk of cardiovascular disease (up to 12 mo)
Wan et al^7^ (2023)	Adult patients with COVID-19 from the UK Biobank; matched to patients without COVID-19	Composite outcome of heart failure, stroke, and coronary heart disease	Increased short- (up to 21 d) and long-term risks of cardiovascular disease and mortality
Daugherty et al^10^ (2021)	Adults with SARS-CoV-2 infection; 3 comparator groups, matched by propensity score.	50 long-term outcomes including coronary heart disease, stroke, congestive heart failure	Increased risk of cardiovascular disease (up to 6 mo)
Zahid et al^11^ (2023)	Adult patients with COVID-19 hospitalization	Myocardial infarction	Incidence at 30 d ∼0.6%
			Age, male, comorbid conditions, lower income associated with higher odds of myocardial infarction

This study also has several limitations that may affect its interpretation and applicability. The use of administrative codes for classifying both COVID-19 infection and the primary outcome could result in misclassification, which may indicate that the overall estimates provided may actually represent the lower bound of real-world MI risk after COVID-19.^18^ The primary outcome is also subject to the competing risk of all-cause death, as patients need to remain alive and at risk in order to face the outcome.^19^^,^^20^ This competing risk of death may also affect the identification of individual risk factors for post-COVID CVD. Furthermore, the outcome assessment did not include additional cardiovascular events and was limited to the 30-day timeframe; as such, the impact of COVID-19 on subsequent, longer-term cardiovascular events is potentially higher (eg, through increased risk of stroke, congestive heart failure, or cardiovascular death). Finally, the authors did not have information on in-hospital characteristics, including the deployment of life-sustaining therapies, intensity of the acute illness, and biomarkers—all of which may be associated with a differential risk of MI after hospital discharge.^21^

All in all, the study by Zahid et al^11^ highlights MI as a relevant downstream short-term complication of COVID-19. While the 30-day incidence is below 1%, this finding is still relevant given the high incidence of COVID-19 infections in the population. From an individual perspective, these results also highlight potential subgroups that may face a higher than average risk of MI (eg, older males with high comorbid burden). The study also showcases some outstanding questions that have yet to be answered by the literature. Future studies should seek to: 1) understand the underpinnings of the association between infection and CVD; 2) identify whether specific infections confer differential cardiovascular risk; 3) better delineate subgroups of patients who remain at higher risk; and 4) identify and evaluate potential mitigation strategies to prevent downstream CVD. Potential avenues for prevention include either targeting pre-existing comorbidities or, alternatively, modifying the inflammatory and coagulopathic consequences of infection. Lastly, while the short-term effects of COVID-19 are comparatively well studied, the long-term effects of COVID-19 on cardiovascular risk profile are also of particular interest and require further study.

## Funding support and author disclosures

The authors have reported that they have no relationships relevant to the contents of this paper to disclose.
